# Are the Latin American recommendations for the management of patients infected with COVID -19 on hemodialysis realistic in health systems with limited resources?

**DOI:** 10.1590/2175-8239-JBN-2020-0059

**Published:** 2020-09-16

**Authors:** Percy Herrera-Añazco, Cristian León Rabanal, Vicente Aleixandre Benites-Zapata

**Affiliations:** 1 Hospital Nacional 2 de Mayo, Lima, Perú.; 2 Universidad Señor de Sipán, Chiclayo, Peru.; 3 Hospital Nacional Cayetano Heredia, Lima, Perú.; 4 Universidad San Ignacio de Loyola, Unidad de Investigación para la generación y síntesis de evidencias en salud, Lima, Perú.

Dear Editor,

In the context of the global epidemic of coronavirus infection (COVID-19), the Latin American Society of Nephrology and Hypertension (SLANH), the Transplant Society of Latin America and the Caribbean (STALYC), and the Pan American Association of Infectious Diseases (API) prepared a statement with recommendations for the management of the COVID-19 epidemic at the regional level for patients with kidney disease^1^. Although it is an important effort, some recommendations are difficult to apply in countries with limited resources like Peru. In our country, despite improvements in the supply of nephrology services^2^, there are structural aspects that prevent compliance with these recommendations and show that our health system is not prepared to face this epidemic.

One of the recommendations suggests that, when there are suspected or confirmed COVID-19 patients, there should be a separation of two meters between patients during hemodialysis (HD) treatment^1^. In Peru, the rules for hiring the HD service in the Ministry of Health (MINSA) require 80 cm as the minimum distance between each HD station^3^. This recommendation is followed by most HD centers in national referral hospitals and by clinics that outsource HD services. There is a structural deficit that means that few hospitals have the possibility to “separate rooms or boxes in isolated conditions” where suspected and infected patients would be dialyzed, as the statement also suggests^1^.

Due to the number of patients on HD, some referral hospitals have been forced to have up to five shifts per day, which would make it impossible to open new dialysis shifts for infected patients, as was also suggested^1^. One of these hospitals is “Dos de Mayo National Hospital” in Lima (HN2M), one of the national referral centers for patients infected with COVID-19 designated by the Peruvian government^4^. This situation is complicated because there are regions in our country where there are no HD centers^2^ and whose patients are referred to hospitals like HN2M^5^, which would make it very difficult to comply with the recommendation to “not transfer patients without alarm symptoms for hospital admission and/or hemodialysis in acute units”^1^. Likewise, the deficit of HD centers and even nephrologists^2^ makes the alternative of home HD or home peritoneal dialysis also not workable in Peru^1^.

At The Peruvian Ministry of Health, an institution that provides health services for low-income citizens in Peru, the patients go to hospitals that not necessarily are close to their home at that time^5^, and the use of public transport is frequent, so the recommendation that patients travel in “individual ambulances or own vehicles” does not fit the reality from Peruvian low-income citizens. We think that there is a situation very similar in some other Latin American countries. 

Although the recommendations shared by SLANH, STALYC, and API are undoubtedly very valuable, local nephrology societies should urgently propose working groups to generate, based on these directive statements, recommendations according to the reality of each region in order to lessen the impact of this pandemic in the vulnerable population of HD patients worldwide and the personnel responsible for their care.
